# Role of NO in Disease: Good, Bad or Ugly

**DOI:** 10.3390/biomedicines12061343

**Published:** 2024-06-18

**Authors:** Anders O. Larsson, Mats B. Eriksson

**Affiliations:** 1 Department of Medical Sciences, Section of Clinical Chemistry, Uppsala University, 751 85 Uppsala, Sweden; anders.larsson@akademiska.se; 2 Department of Surgical Sciences, Section of Anaesthesiology and Intensive Care Medicine, Uppsala University, 751 85 Uppsala, Sweden; 3 NOVA Medical School, New University of Lisbon, 1099-085 Lisbon, Portugal

This Special Issue of *Biomedicines* (https://www.mdpi.com/journal/biomedicines/special_issues/NO_in_Disease), accessed on 30 May 2024, focuses on the role of nitric oxide in disease, attempting to demonstrate some of its “Good, Bad, or Ugly” effects. The Editors wish to thank the authors for their valuable contributions, and we are happy to present valuable studies on nitric oxide (NO) from multiple perspectives. NO is present in the airways, and its relationships with both chronic obstructive pulmonary disease and rheumatoid arthritis are highlighted. NO donations may also have therapeutic potential in bladder cancer. Furthermore, the biological sensing of NO using a ruthenium-based sensor may have great utility in atherosclerosis. NO in human breast milk, as determined via total nitrite and nitrate concentrations, is more abundant in the first 30 days than at day 60 postpartum; this may have implications for successful and unsuccessful breastfeeding. This Special Issue also contains five review articles, three of which indicate the importance of NO in various tissues and its relation to diseases; the potential effects of NO are reviewed in this context. Due to the SARS-CoV-2 pandemic, considerable interest has been paid to inhaled NO (iNO) and its ability to reduce inflammatory lung injury, lower pulmonary vascular resistance, and enhance ventilation/perfusion matching.

This Special Issue emphasizes the importance of NO and presents new data through various overviews that collate recent updates on NO.

In 1998, Robert Furchgott, Louis Ignarro, and Ferid Murad were awarded the Nobel Prize in Physiology or Medicine for their significant discovery of nitric oxide (NO) as a signaling molecule in the cardiovascular system. According to Alfred Nobel’s will, a maximum of three persons can share the prize [1]. Therefore, Salvador Moncada was not awarded the Nobel Prize (a decision that has been criticized, even by Robert Furchgott) [2].

This highly potent two-atom radical containing an unpaired electron exhibits a wide range of physiological activities. NO has played a crucial role in evolution, from fungi to mammals, acting as both an intercellular and intracellular messenger in invertebrates [3].

NO is an essential biological mediator in the living organism that is biosynthesized from L-arginine using NADPH and molecular oxygen, a reaction catalyzed by enzymes termed nitric oxide synthases, consisting of different subtypes depending on the tissue type. Nitrite can act as a substrate for NOS-independent generation of NO in vivo, and such reduction can occur systemically in both blood and tissues [4,5]. NO has a short biological lifetime that can be counted in seconds (or even less) depending on its presence in intravascular/extravascular tissues [6]. In blood, NO rapidly reacts with oxygenated hemoglobin, forming its metabolites, nitrate and nitrite. The reaction rate of NO in aqueous solutions of with oxygen and hemoglobin follows second-order kinetics, i.e., the rate of NO’s disappearance is proportional to the square of the concentration of NO. Thus, NO is not likely to act as a circulating humoral substance [7]. NO produced in the endothelial cells of blood vessels signals the surrounding smooth muscle to relax, leading to vasodilation and allowing for the biological activity of endothelium-derived relaxing factor and a subsequent increase in blood flow [8,9]. NO is also an important messenger in the central nervous system, where it facilitates cell communication. Glutamate, a neurotransmitter, starts a reaction that forms NO. NO regulates several important functions in the central nervous system, including processes associated with mood disorders [10,11].

Since NO among several other options is a vasodilator able to reduce both systemic and pulmonary blood pressure [12], various drugs have been designed to activate NO signaling and enhance NO bioavailability as beneficial cardiovascular effects; alternatively, by contrast, they may attenuate NO inactivation through reactive oxygen species exerting antioxidant effects. More recently, the products of NO oxidation, nitrite and nitrate, have been acknowledged as sources of NO after recycling back to NO. Activation of the nitrate–nitrite–NO pathway may generate NO from both anions and induce antihypertensive effects. Interestingly, human arterial blood added to a ruthenium-based NO sensor complex may be utilized as a point-of care test for early detection of unstable coronary plaque and monitoring of NO-related cardiovascular disease [13].

Furthermore, endogenous NO continuously regulates pulmonary and systemic circulations in several species (including humans), as evidenced by the fact that NOS inhibition increases pulmonary and systemic vascular resistance. Additionally, endogenous NO modifies hypoxic vasoconstriction. Inhaled NO is a selective pulmonary vasodilator; it rapidly diffuses across the alveolar–capillary membrane into the pulmonary vessels, where NO activates guanylate cyclase. Since inhalation of NO is a prerequisite for such an effect on the pulmonary vascular bed, the risk of ventilation/perfusion mismatch and pulmonary shunting is diminished. NO may decrease pulmonary arterial pressure and improve oxygenation and has therefore been approved by the FDA for treatment of hypoxic newborns affected by persistent pulmonary hypertension. More recently, the outbreak of SARS-CoV-2, a respiratory infectious disease that causes both pulmonary and cardiovascular complications, has exposed new indications for NO therapy. Endothelial dysfunction, increased pulmonary vascular permeability, and the formation of pulmonary venous thrombi frequently accompany SARS-CoV-2 infection and contribute to the development of pulmonary artery hypertension. Inhaled NO has the ability to counteract several of these deleterious effects of COVID-19. In a multicenter phase II trial, high-dose inhaled nitric oxide improved arterial oxygenation in adults with acute hypoxemic respiratory failure due to COVID-19. Whether inhaled NO may serve as an adjunct therapy against bacterial, viral, and fungal infections remains to be elucidated [14,15,16,17,18].

NO is synthesized during sepsis and septic shock, conditions that feature increased levels of NO and lowered blood pressure, the latter leading to subsequent impairment of organ perfusion. Several clinical trials have attempted to modulate the formation of inducible nitric oxide synthase (iNOS) from NO through treatment with nitric oxide synthase (NOS) inhibitors, of which L-NAME has been the most extensively studied. Contrary to what could be expected, L-NAME turned out to increase mortality in sepsis patients by increasing the number of cardiac and pulmonary adverse events. Asymmetric dimethylarginin (ADMA) is a direct endogenous NOS inhibitor that exerts microvascular dysfunction and proinflammatory and prothrombotic conditions in the endothelium, and there is a relationship between plasma levels of ADMA and mortality in sepsis patients. Hence, the lowering of ADMA has been suggested as a potential therapeutic approach to reduce organ damage and mortality in sepsis. NO may cause methemoglobinemia, a potentially life-threatening condition, since this form of hemoglobin cannot bind oxygen. This condition can be treated with methylene blue, which acts by blocking the enzyme guanylate cyclase, thereby reducing excessive nitric oxide production, counteracting its vasorelaxant effect, and increasing blood pressure during sepsis. Patients with sepsis and septic shock treated with methylene blue exhibit reduced time until vasopressor discontinuation, reduced length of stay in the intensive care unit, and reduced days on mechanical ventilation as compared to placebo. In a retrospective study evaluating the effect of methylene blue in shock, reduced 28-day mortality in critically ill patients was noted when methylene blue was administered as a bolus followed by a continuous infusion [19,20,21].

NO is a free radical that along with other free radicals contributes to the body’s defense against micro-organisms. NO may also scavenge other free radicals and reacts in vivo with superoxide, thereby forming peroxynitrite, a highly reactive compound that can generate nitrogen dioxide and carbonate radicals upon reaction with carbondioxide. Excessive formation of peroxynitrite may contribute to numerous adverse events, including DNA damage and the disruption of cell membranes. Peroxynitrite is involved in several diseases but may also, at high concentrations, protect against microbes. At low concentrations, peroxynitrite exerts protective mechanisms in several organ systems [22]. Abundant production of NO can induce the release of inflammatory cytokines and also trigger oxidative stress, factors involved in the pathogenesis of several immunopathologies including diabetes, graft-versus-host reaction, rheumatoid arthritis, systemic lupus erythematosus, experimental autoimmune encephalomyelitis, and multiple sclerosis [23].

Thus, due to the multiple physiological and pathophysiological roles of NO, this Janus-faced molecule can act as a double-edged sword, exerting good, bad, and ugly effects.
